# Feasibility and clinical effectiveness of pre-hospital prothrombin complex concentrates (Octaplex/Beriplex) in anticoagulated patients with traumatic intracranial haemorrhage

**DOI:** 10.1186/1757-7241-23-S2-A14

**Published:** 2015-09-11

**Authors:** Ben Brooks, Suzanne Kellett, Simon Hughes

**Affiliations:** 1 Faculty of Medicine, University of Southampton, Southampton, UK; 2 Shackleton Department of Anaesthetics, University Hospital Southampton Foundation Trust, Southampton, UK

## Background

Incidence and mortality from traumatic intra-cranial haemorrhage (ICH) is increased in patients on warfarin [1]. Prothrombin Complex Concentrates (PCC) rapidly reverse warfarin anti-coagulation and have been shown to reduce haematoma expansion and mortality in warfarinised patients sustaining ICH [2].

We aimed to determine the feasibility of pre-hospital use of PCCs by physicians and their clinical effectiveness in warfarinised patients sustaining traumatic ICH.

## Method

Patients were identified from the Trauma Audit and Research Network (TARN) database. We recorded demographic data, transfer method, injury mechanism and severity, time to blood sampling and reversal of anticoagulation, effectiveness of PCCs and patient outcome data.

## Results

197 patients aged over 60 with traumatic ICH were admitted to University Hospital Southampton between April 2012 and May 2014. Mean age was 79 (range 61-98). More patients, 32/197 (16%), were anti-coagulated with warfarin, than other study populations (9%) [3]. One patient arrived by helicopter.

International Normalised Ratio (INR) was obtained within 8 hours of admission in 80% of cases. Failure to achieve this target was usually a result of inadequacy of samples sent. A small number of patients died before samples could be obtained. 11% (21/197) of patients had an INR ≥2.0, and were reversed with PCC and vitamin K. Post-reversal INR ≤1.5 was not achieved in two patients (INR = 1.6). Overall mortality for warfarinised patients (25%) was higher than their non-anti-coagulated counterparts (15%).

## Discussion

Only one anti-coagulated patient with traumatic ICH was treated by a pre-hospital physician. The majority of these patients received timely and effective reversal of their anticoagulation in-hospital following current guidelines. Notably, only 11% had an INR >2.

Pre-hospital PCC use requires investment in portable near-patient testing, interpretation of results and prescription of blood products. Our results support rapid transfer to hospital and timely, preferably near-patient, testing rather than investment in pre-hospital PCC use.
